# Characteristics associated with quality of life among people with drug-resistant epilepsy

**DOI:** 10.1007/s00415-017-8512-1

**Published:** 2017-05-26

**Authors:** Leone Ridsdale, Gabriella Wojewodka, Emily Robinson, Sabine Landau, Adam Noble, Stephanie Taylor, Mark Richardson, Gus Baker, Laura H. Goldstein, SMILE Team, Paul McCrone, Matthew Walker, Dora Lozsadi, Bridget MacDonald, Jennifer Quirk, Robert S. Delamont, Michalis Koutroumanidis, Lina Nashef, Nandini Mullatti, Asra Siddiqui, Hannah Cock, Fergus Rugg-Gun, Dominic Heaney, John Duncan, Robert Elwes, Alison McKinlay, Carly Pearson, Sarah Feehan, Iris Mosweu, Ray Chaudhuri

**Affiliations:** 1 Department of Basic and Clinical Neuroscience, Institute of Psychiatry, Psychology and Neuroscience, Academic Neuroscience Centre, King’s College London, Rm A2.06, Denmark Hill, PO Box 57, London, SE5 8AF UK; 2 Institute of Psychology, Health and Society, University of Liverpool, Liverpool, UK; 3 Centre for Primary Care and Public Health, Bart’s and the London School of Medicine and Dentistry, Queen Mary University of London, London, UK

**Keywords:** Epilepsy, Quality of life, Anxiety, Depression, Self-mastery, Stigma

## Abstract

Quality of Life (QoL) is the preferred outcome in non-pharmacological trials, but there is little UK population evidence of QoL in epilepsy. In advance of evaluating an epilepsy self-management course we aimed to describe, among UK participants, what clinical and psycho-social characteristics are associated with QoL. We recruited 404 adults attending specialist clinics, with at least two seizures in the prior year and measured their self-reported seizure frequency, co-morbidity, psychological distress, social characteristics, including self-mastery and stigma, and epilepsy-specific QoL (QOLIE-31-P). Mean age was 42 years, 54% were female, and 75% white. Median time since diagnosis was 18 years, and 69% experienced ≥10 seizures in the prior year. Nearly half (46%) reported additional medical or psychiatric conditions, 54% reported current anxiety and 28% reported current depression symptoms at borderline or case level, with 63% reporting felt stigma. While a maximum QOLIE-31-P score is 100, participants’ mean score was 66, with a wide range (25–99). In order of large to small magnitude: depression, low self-mastery, anxiety, felt stigma, a history of medical and psychiatric comorbidity, low self-reported medication adherence, and greater seizure frequency were associated with low QOLIE-31-P scores. Despite specialist care, UK people with epilepsy and persistent seizures experience low QoL. If QoL is the main outcome in epilepsy trials, developing and evaluating ways to reduce psychological and social disadvantage are likely to be of primary importance. Educational courses may not change QoL, but be one component supporting self-management for people with long-term conditions, like epilepsy.

## Introduction

Drug management enables the majority of people with epilepsy (PWE) to control their seizures, but in about 40% seizures persist [1, 2]. Long-term persisting seizures expose PWE to further risk of psychological and social disadvantage, as well as to premature death [3–5]. Barriers to seizure control include: severe brain pathology, psychological co-morbidity [6], social disadvantage [7, 8], and lack of provision of self-management advice and support [9]. Some of these barriers may be amenable to change through psychological, social and educational interventions [10, 11]. None have been tested in the United Kingdom (UK) by means of a large randomized controlled trial.

In this context, we aimed to recruit a large group of PWE with persistent seizures from specialist clinics, and test the effectiveness and cost-effectiveness of a 2-day self-management education course in an RCT [12]. The UK National Institute of Clinical Excellence (NICE) and National Institute of Health Research (NIHR) require evidence of change following complex interventions, with the primary outcome being quality of life (QoL) [13]. Many instruments have been used to measure QoL in randomized controlled trials with PWE, some more generic and others adapted specifically for epilepsy, such as the QoL in Epilepsy Scale (QOLIE). A study using a non-epilepsy-specific QoL measure had been unable to show an improvement in scores in patients attending self-management courses [14]. However, two studies using epilepsy-specific QoL measures had shown improvements in the intervention group [15, 16]. In one, some domains of QoL improved immediately after the intervention, but benefits did not persist at 6 months [15]. In the other, there had been co-interventions including monthly appointments with a pharmacist [16]. It, therefore, remains to be seen whether an epilepsy-specific QoL measure would improve following a stand-alone self-management education course after 1 year.

There is little evidence about QoL among UK PWE [17, 18]. From international evidence we knew QoL in epilepsy is multidimensional, and consistently associated with psychological and epilepsy status [19–21]. Social characteristics such as stigma, perceived self-mastery, and the effect of self-management education interventions have not been measured consistently in international studies [8, 23, 24]. In this context, and prior to carrying out an evaluation of the effectiveness of a self-management course on QoL, we aimed to answer the questions: (1) what are the clinical, psychological and social characteristics of UK adults with persistent seizures?; (2) to what extent do the individual domains of the QoL instrument correlate with the total measure?; (3) to what extent are clinical, psychological and social characteristics, which underlie constructs of QoL, associated with QoL?

## Methods

As is recommended we published the trial design and analysis methods early on [12, 13].

### Eligibility criteria

To participate in the trial, patients had to: have a diagnosis of epilepsy (all epilepsy syndromes) documented by a specialist, have had at least two self-reported seizures in the previous 12 months, be currently prescribed anti-epileptic drugs, be aged ≥16 years, be able to provide informed consent, be able to participate in a 2-day epilepsy self-management course, and be able to complete questionnaires in English [12]. Exclusion criteria were having non-epileptic seizures only, having seizures related to an acute illness or substance overuse, having a serious psychiatric condition or terminal illness, and participating in other epilepsy-related studies [12].

### Recruitment

Participants were recruited from epilepsy clinics at eight hospitals in South East England. In the context of frequent memory problems reported among PWE [25], likely lack of harm from this educational intervention, and to maximise participation, recruitment was by an opt-out process [26]. Individuals had two opportunities to opt out by returning a paper slip by pre-paid post: (1) prior to medical note screening and (2) once deemed eligible after medical note screening, and prior to contact by researcher. Thus, patients could opt out of the recruitment process without having to speak to clinical staff or a researcher. When being contacted by a researcher, patients could still verbally decline to enrol in the study. Participants enrolled into the study were subsequently asked to give written informed consent at a face-to-face meeting with a researcher.

The study was approved by the National Research Ethics Service Committee London—Fulham (REC reference 12/LO/1962). Trial registration: ISRCTN57937389.

### Assessments

Primary and secondary outcomes were used according to general specifications made by our national funding agency, with flexibility offered about specific measures used. The information was obtained by means of a composite questionnaire using validated assessments, which included the following:

#### Quality of life

A heath-related epilepsy-specific QoL scale was used, the QoL in Epilepsy Scale (QOLIE). The QOLIE-31 has different versions. QOLIE-31-P [22] is a modified version of the QOLIE-31 [27] with added patient-specific weightings. It contains seven domains reflecting aspects affected by living with epilepsy: energy, mood, daily activity, cognition (including memory), medication effects, seizure worry, and overall QoL. Scores for domains and for total QOLIE-31-P were calculated according to existing methods and can range from 0 to 100 [22], with higher scores indicating better QoL. QoL was included as the primary outcome because the funding agency specified this.

#### Demographics

These included age, gender, ethnicity, education, employment, living arrangements, marital status, and the Index of Multiple Deprivation (IMD). The IMD measures the level of deprivation, using participants’ postcode and data from the UK Office of National Statistics [28] which are normalized so a ‘normal’ distribution includes 20% of the population values in each quintile.

#### Clinical and psychological characteristics

These included years since diagnosis, number of seizures in the previous year [3], date of last seizure, and physical/psychiatric medical history. Current psychological distress was measured by the 14-item Hospital Anxiety and Depression Scale (HADS) which classifies cases or borderline cases of anxiety and depression with cut-offs at 0–7 for normal, 8–10 for borderline, ≥11 for case estimates [29].

#### Social and self-management characteristics

Self-management assessments included perceived self-mastery over epilepsy using an epilepsy-specific scale [30]. Scores range from 6 to 24 with a higher score representing greater perceived self-mastery. Medication adherence was recorded using the ten-item Medication Management subscale from the Epilepsy Self-Management Scale [31]. Scores range from 10 to 50, with greater scores indicating better adherence to medication plans.

To measure the social impact of epilepsy, we assessed perceived stigma with the Stigma of Epilepsy Scale. It includes three statements: “Because of epilepsy, (1) other people are uncomfortable with me, (2) treat me as inferior, and (3) prefer to avoid me”. It is scored on a Likert-type four-point scale: “not at all”, “yes, maybe”, “yes, probably” and “yes, definitely”, which is scored from 0 to 9 and categorized as not stigmatized (score of 0), mild-moderate (1–6) and highly stigmatized (7–9) [23].

### Statistical analysis

The statistical analysis plan for the trial has been described [13]. To characterize our baseline sample, demographic, social and clinical data are described using relevant summary statistics. To describe the QOLIE-31-P instrument, the total QOLIE-31-P score was used as the dependent variable to test associations with each of the scale’s individual domains. Each pairwise combination of the individual domains was compared to each other using Pearson’s correlation to determine whether they assess similar components of QoL. Similarly, we tested whether individual domains were correlated with HADS anxiety and depression scores.

To investigate associations between total QOLIE-31-P score and other baseline measures such as demographics, simple linear regression analyses were performed. Associations between total QOLIE-31-P score and other continuous measures are represented by Pearson’s correlation coefficients (*r*), as above. To assess an association with a categorical variable, dummy variables were created to represent the effect of the factor and to select a reference category, and then *F* tests were used for the combined effect of the respective variables. To aid interpretation of such effects, marginal means (MM) of total QOLIE-31-P were estimated for each level of the factor variable, and similarly the MMs were estimated for each quartile of the continuous variables. Coefficients and MMs from the simple linear regression are provided with 95% confidence intervals (CIs), along with *p* values of significance tests. For each scale, a category was chosen as a reference for comparative purposes (“ref”). For example, when looking at gender, the category “male” was chosen as a reference to which the category “female” was compared.

## Results

### Participant recruitment

**Fig. 1:**
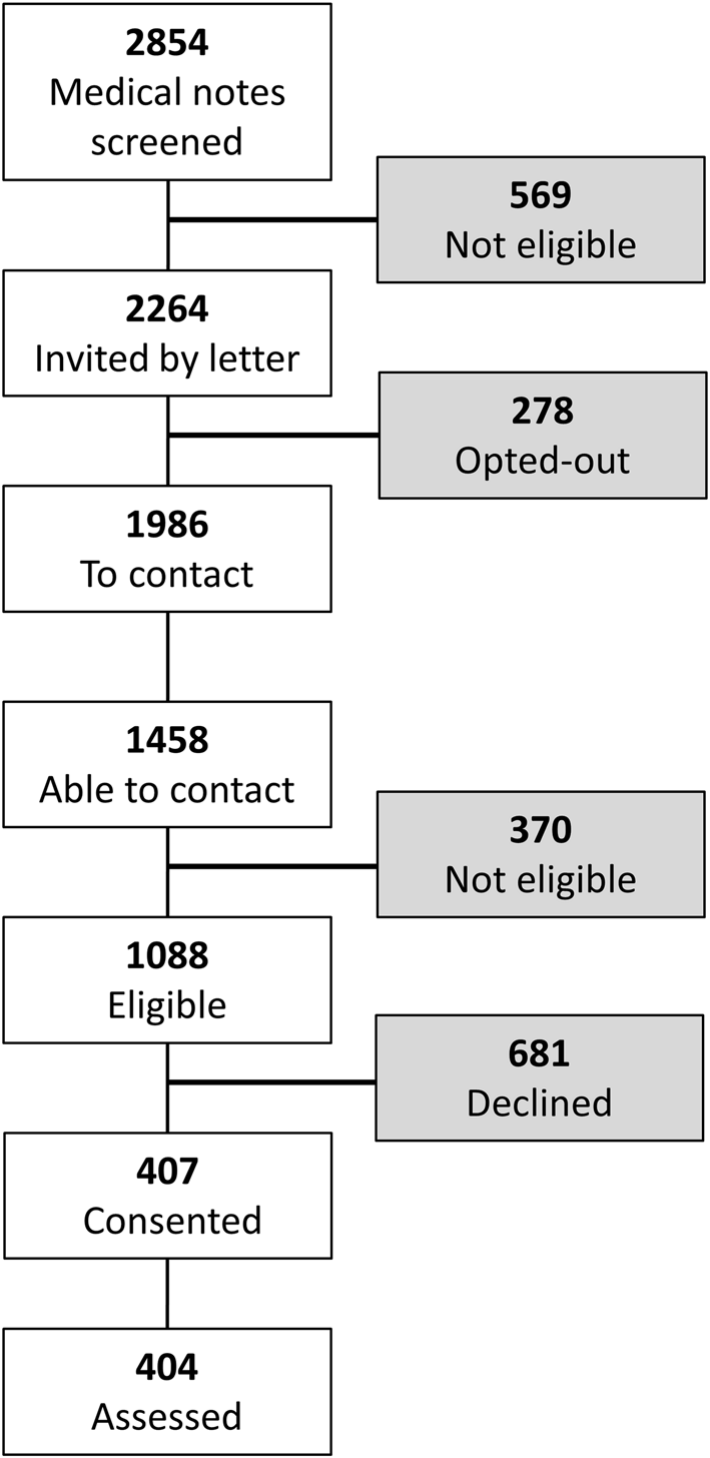
Participant recruitment process. In the first stage of opt-outs, an invitation letter was sent by consultants to patients who had attended their clinic in the past year (not illustrated here), with a 3-week window to opt out from future contact. Medical notes of patients not opting out were screened for eligibility criteria. Ineligibility at this stage was primarily not having at least two seizures in the previous year. In combination, the opt-out stages involved two invitation letters and two opportunities to opt out resulting in 1986 participants remaining in the pathway. Three attempts were made to contact patients and research workers contacted 1458 patients. Eligibility was assessed once again. Ineligibility at this stage was primarily not having two seizures within the past year or living outside the study’s catchment area. 681 patients verbally declined to participate and finally 404 participants consented and assessed for baseline data. These participants were randomized into treatment groups for the study. *Grey boxes* indicate the number of individuals who opted out/declined or were not eligible for the study

Figure 1 illustrates the pathway for those not opting out of the recruitment process and who had their medical records screened. From a final group of 1088 eligible patients, 407 participants enrolled in this study, with 404 completing assessments, representing a 37% recruitment rate.

### Description of sample of PWE

#### Demographics

Demographic characteristics of the sample of people with poorly controlled epilepsy are described in Table 1. The average age of participants was 41.7 years (SD 14.1) with 54.2% being female, and three-quarters white (75.3%). Almost one-third (31.5%) were educated to university degree level or above. However, almost one half (49.2%) of the total had no paid work. Most of the PWE lived with at least one other person (75.5%) and 51% were single. A higher than national proportion of this group of PWE lived in the most deprived areas, 60.7% (versus 40%) in IMD quintiles 4 and 5.

**Table 1:** Participant demographics

Factor	Level	*n* = 404
Age mean (SD) [range]		41.7 (14.1) [16, 85]
Gender *n* (%)	Female	219 (54.2)
	Male	185 (45.8)
Ethnicity *n* (%)	White	304 (75.3)
	Mixed/multiple ethnic groups	40 (9.9)
	Asian/Asian British	18 (4.5)
	Black/African/Caribbean/Black British	33 (8.2)
	Other	9 (2.2)
Education^a^ *n* (%)	No formal qualifications	61 (15.1)
	Secondary	131 (32.4)
	Further education	85 (21.0)
	Higher education	127 (31.5)
Employment (≤64 years) *n* (%) (*n* = 382)	Specifically employed or student	194 (50.8)
	Not employed	188 (49.2)
Living arrangements *n* (%)	Living with others	305 (75.5)
	Living alone	95 (23.5)
Marital status *n* (%) (*n* = 402)	Single	205 (51.0)
	Steady relationship but not cohabiting	44 (11.0)
	Married/living with partner	153 (38.1)
IMD quintiles *n* (%)	1 (least deprived)	39 (9.7)
	2	56 (13.9)
	3	64 (15.8)
	4	136 (33.7)
	5 (most deprived)	109 (27.0)

^a^Further education: any qualification obtained post-secondary level, excluding university level. Higher education: Bachelor’s degree and higher

#### Clinical and psychological characteristics

Participants had been diagnosed with epilepsy for a median of 18 years (range 1–66). This group of PWE reported frequent seizures, with 69.3% having 10 or more per year. The median number of days since their last seizure was 34 days (Table 2). Just under half (45.9%) reported co-morbidity, including 13.2% reporting a prior ‘psychiatric condition’. HADS scores suggested that a larger proportion, 53.6%, had current borderline or case levels of anxiety, and 28% had current borderline or case depression (Table 2).

**Table 2:** Clinical, psychological, social and self-management assessments of PWE

Scale	Result
Years since epilepsy diagnosis, mean (SD); median,[range] (*n* = 403)	21.2 (15.5); 18 [1, 66]
Seizure frequency *n* (%), (*n* = 404)	
1–3	49 (12.1)
4–5	51 (12.6)
7–9	24 (5.9)
10+	280 (69.3)
Days since last seizure (*n* = 383), median (IQR) [range]	34 (18, 63) [1, 351]
Co-morbidities *n* (%)	
No	219 (54.2)
Yes, another medical condition	132 (32.7)
Yes, psychiatric condition	20 (5.0)
Yes, both medical and psychiatric conditions	33 (8.2)
HADS anxiety *n* (%), (*n* = 403)	
Normal (0–7)	187 (46.4)
Borderline (8–10)	79 (19.6)
Case (≥11)	137 (34.0)
HADS depression *n* (%), (*n* = 403)	
Normal (0–7)	290 (72.0)
Borderline (8–10)	71 (17.6)
Case (≥11)	42 (10.4)
Stigma of epilepsy *n* (%), (*n* = 401)	
Not stigmatized (0)	148 (36.9)
Mild-moderate (1–6)	203 (50.6)
Highly stigmatized (7–9)	50 (12.5)
Self-Mastery of Epilepsy Scale, mean (SD) [range], (*n* = 399)	14.1 (3.3) [6, 24]
Medication Adherence Scale, mean (SD) [range], (*n* = 399)	45.4 (4.8) [16.7, 50]

#### Social and self-management characteristics

A high proportion (63.1%) felt some level of epilepsy-related stigma (Table 2). The mean score for the perceived Self-Mastery of Epilepsy Scale was 14.1 (SD 3.3), indicating that on average they felt they had some control over their condition, with room for improvement. The Medication Adherence Scale suggested that they felt they followed their medication plan well, as the average score was 45.4 out of a maximum of 50 (Table 2).

#### Quality of life

Compared to a maximum possible score of 100, the mean score on the QOLIE-31-P scale was 66.0 (SD 14.2), with a wide range from 24.8 to 98.5. When patient-specific weighting is removed, the QOLIE-31 mean score was 62.0 (SD 15.6), ranging from 24.5–97.6. The seven subscales of QOLIE-31-P reflecting domains affected by living with epilepsy are presented in Table 3a. The lowest subscale score was for energy, followed by cognition (which includes memory) and seizure worry. Table 3a shows how each of the QOLIE-31-P subscales contributed a similar amount to the total score, with strong pairwise correlation coefficients, ranging from 0.63 to 0.71. This suggests the QOLIE-31-P is not dominated by a particular subscale. The correlations between subscales were weaker, suggesting they are indeed measuring different domains. Table 3b shows that HADS anxiety is particularly associated with mood and seizure anxiety domains, whilst HADS depression is associated with mood, energy and daily activity. Thus, current psychological distress is associated with participants’ perception that QoL is reduced.

**Table 3:** Pearson’s correlation coefficients (*r*) between (a) all pairwise combinations of total and domain sub-scores of QOLIE-31-P and (b) total and domain sub-scores of QOLIE-31-P with HADS

(a)								
QOLIE-31-P	Score mean (SD) [range]	Total	Energy	Mood	Daily activity	Cognition	Medication effects	Seizure worry
Total	66.0 (14.2) [24.8, 98.5]							
Energy	53.4 (18.1) [16.7, 100]	0.68						
Mood	67.2 (17.6) [16.7, 100]	0.67	0.53					
Daily activity	65.2 (23.5) [19.3, 100]	0.71	0.42	0.39				
Cognition	59.2 (23.6) [18.6, 100]	0.68	0.44	0.44	0.43			
Medication effects	67.7 (23.8) [21.7, 100]	0.68	0.33	0.27	0.47	0.38		
Seizure worry	61.8 (21.7) [24.0, 100]	0.63	0.34	0.40	0.44	0.40	0.43	
Overall QoL	62.6 (18.2) [10.0, 100]	0.67	0.55	0.65	0.43	0.44	0.25	0.38

(b)		
Scale	Correlation coefficient (HADS-A)	Correlation coefficient (HADS-D)
QOLIE-31-P scale (*n* = 400)	−0.63	−0.66
QOLIE-31-P subscales		
Energy (*n* = 402)	−0.46	−0.57
Mood (*n* = 402)	−0.67	−0.60
Daily activity (*n* = 400)	−0.40	−0.51
Cognition (*n* = 402)	−0.42	−0.46
Medication effects (*n* = 399)	−0.35	−0.37
Seizure worry (*n* = 401)	−0.51	−0.35
Overall quality of life (*n* = 400)	−0.45	−0.56

### Associations of demographic, clinical and psycho-social factors with QOLIE-31-P

#### Demographics

Total QOLIE-31-P scores were found to be moderately associated with several demographic factors (Table 4). Females had lower scores than males (females MM: 64.2; males MM: 68.2). Less education (no formal qualifications MM: 61.8; higher education MM: 68.3) and not being employed (not employed MM: 62.0; employed MM: 69.5) were associated with lower QOLIE-31-P scores.

**Table 4:** Participant characteristics and associations with QOLIE-31-P

Baseline characteristics (categorical)		Marginal mean (95% CI)	Coefficient (95% CI)	*p* value
Scale	Level			
Gender (*n* = 400)	Male (ref)	68.2 (66.2, 70.3)	–	0.0043
	Female	64.2 (62.3, 66.1)	−4.1 (−6.8, −1.3)	
Highest level of education achieved (*n* = 400)	Higher education (ref)	68.3 (65.9, 70.8)	–	0.0096
	Further education	67.8 (64.8, 70.8)	−0.5 (−4.4, 3.4)	
	Secondary	64.6 (62.2, 67.1)	−3.7 (−7.1, −0.2)	
	No formal qualifications	61.8 (58.2, 65.3)	−6.6 (−10.9, −2.2)	
Employment status (≤64 years) (*n* = 379)	Employed or student (ref)	69.5 (67.5, 71.4)	–	<0.001
	Not employed	62.0 (60.0, 64.0)	−7.5 (−10.3, −4.7)	
Years since epilepsy diagnosis (*n* = 403)	32 years	67.1 (65.4, 68.8)	0.1 (0.01, 0.2)	0.037
	18 years	65.7 (64.3, 67.2)		
	8 years	64.8 (62.9, 66.6)		
	1 year	64.1 (61.8, 66.4)		
Seizure frequency in previous 12 months (*n* = 400)	1–3 times (ref)	73.6 (69.7, 77.5)	–	<0.001
	4–6 times	68.8 (64.9, 72.6)	−4.8 (−10.3, 0.7)	
	7–9 times	69.3 (63.7, 74.8)	−4.3 (−11.1, 2.5)	
	10+ times	64.0 (62.3, 65.6)	−9.6 (−13.9, −5.4)	
Co-morbidity (*n* = 400)	No (ref)	68.5 (66.6, 70.3)	–	<0.001
	Yes, another medical condition	65.0 (62.7, 67.4)	−3.4 (−6.4, −0.4)	
	Yes, psychiatric condition	61.5 (55.4, 67.6)	−7.0 (−13.4, −6.4)	
	Yes, both medical and psychiatric conditions	56.8 (52.1, 61.6)	−11.6 (−16.7, −6.6)	
HADS anxiety (*n* = 399)	Normal (ref)	74.4 (72.7, 76.1)	–	<0.001
	Borderline	63.7 (61.1, 66.3)	−10.7 (−13.8, −7.6)	
	Case	56.0 (54.1, 58.0)	−18.4 (−21.0, −15.8)	
HADS depression (*n* = 399)	Normal (ref)	70.8 (69.4, 72.1)	–	<0.001
	Borderline	58.1 (55.4, 60.8)	−12.7 (−15.7, −9.6)	
	Case	47.2 (43.6, 50.7)	−23.6 (−27.4, −19.8)	
Stigma of epilepsy (*n* = 397)	Not stigmatized (ref)	71.6 (69.4, 73.8)	–	<0.001
	Mild-moderate	63.9 (62.1, 65.8)	−7.7 (−10.6, −4.8)	
	Highly stigmatized	58.9 (55.1, 62.6)	−12.8 (−17.1, −8.4)	
Self-Mastery of Epilepsy Scale (*n* = 396)	16 self-mastery score	70.0 (68.6, 71.4)	2.1 (1.7, 2.5)	<0.001
	14 self-mastery score	65.9 (64.7, 67.1)		
	12 self-mastery score	61.7 (60.3, 63.1)		
	6 self-mastery score	49.2 (46.0, 52.4)		
Medication Adherence Scale (*n* = 399)	48 med adherence score	66.8 (65.2, 68.3)	0.3 (0.05, 0.6)	0.023
	46 med adherence score	66.1 (64.7, 67.5)		
	43 med adherence score	65.1 (63.5, 66.6)		
	16 med adherence score	55.9 (47.3, 64.6)		

*(ref)* refers to the category used as a reference for comparison

#### Clinical and psychological characteristics

**Fig. 2:**
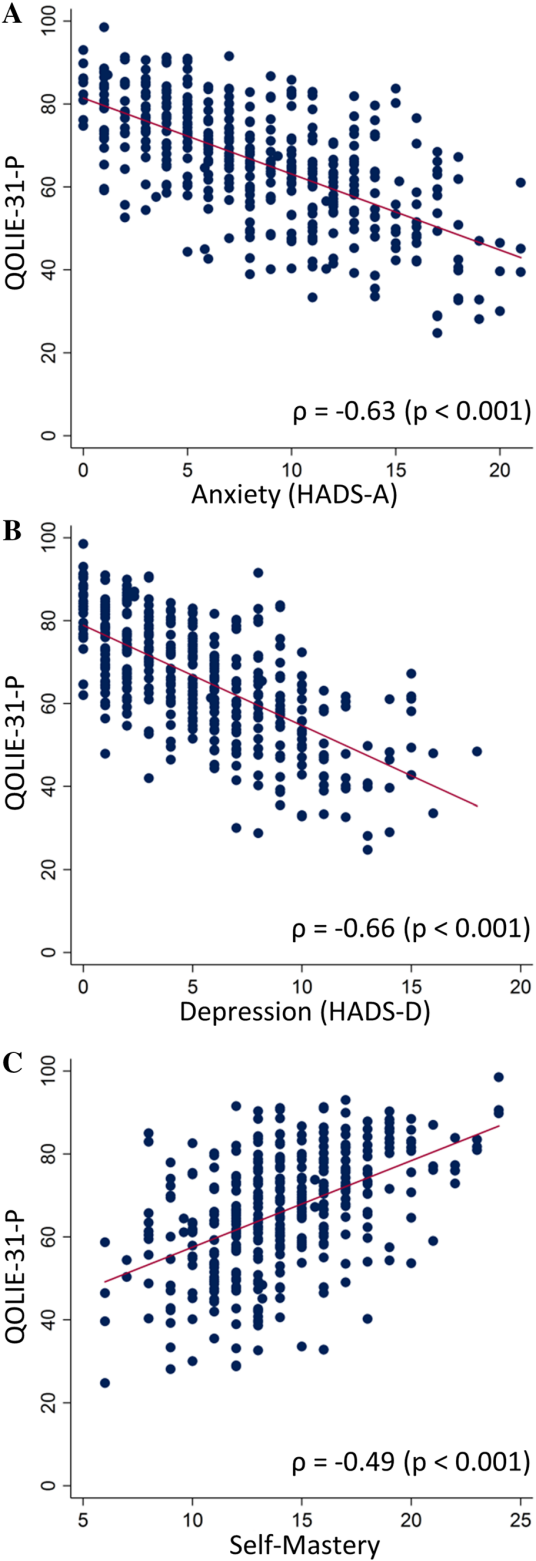
Relationships between quality of life, psychological and self-management assessments in people with epilepsy. Quality of life in epilepsy was measured by QOLIE-31-P. **a** Anxiety, measured by HADS, was significantly associated with total QOLIE-31-P scores (*r* = −0.63, *p* < 0.001, *n* = 400). **b** Depression, measured by HADS, was significantly associated with total QOLIE-31-P scores (*r* = −0.66, *p* < 0.001, *n* = 400). **c** Self-mastery over epilepsy was significantly associated with total QOLIE-31-P scores (*r* = 0.49, *p* < 0.001, *n* = 399). *Red line* represents the fitted simple regression model

A more recent diagnosis of epilepsy (*p* = 0.037) and a higher seizure frequency, specifically with 10 or more seizures in the past year (≥10 seizures MM: 64.0; 1–3 seizures MM: 73.6), were associated with a moderately lower total QOLIE-31-P score. Reporting prior co-morbidity, especially psychiatric, was associated with lower QoL (both medical and psychiatric MM: 56.8; no co-morbidity MM: 68.5). Current borderline or case scores for anxiety or depression determined by HADS were associated with the greatest reductions in QOLIE-31-P scores (no anxiety MM: 74.4; anxiety case MM: 56; no depression MM: 70.8; depression case MM: 47.2). Pairwise correlations of current depression and anxiety HADS scores with total QOLIE-31-P showed that they were closely associated (*ρ* = −0.66, and *ρ* = −0.63, respectively) (Fig. 2a, b).

#### Social and self-management characteristics

Table 4 shows that lower scores on the Self-Mastery Scale were associated with much lower total QOLIE-31-P scores (self-mastery—highest quartile MM: 70; lowest MM: 49.2). Felt stigma (no stigma MM: 71.6; highly stigmatized MM: 58.9) and less medication adherence were also associated with lower total QOLIE-31-P scores. Pairwise correlations of self-mastery and stigma scores with total QOLIE-31-P show their association (*ρ* = 0.49, *p* < 0.001 (Fig. 2c) and *ρ* = −0.31, *p* < 0.001).

## Discussion

### Summary of findings

Due to the restrictions on data that can be legally collected from non-participants, we are unable to evaluate characteristics of those not consenting to the study. Compared to the population of London, our study group was older than the average of 34.0 years, with more white ethnicities, and a greater proportion living in areas of high deprivation [32]. Our group had a higher proportion of people that were single (43.8% vs national statistics: 33.9%) and living with others (75.5% vs national statistics: 60.6%) [33]. Our group of PWE was relatively highly educated with 51.3% having post-secondary qualifications which is close to national figures of 62.6%. Yet despite this, 49.2% were unemployed. Figures for London, UK show 28.5% of people not in work (unemployed and economically inactive between 16 and 64) [34]. Thus, in comparison to general population statistics, the members of our group of PWE with continuing seizures experiences more unemployment, live more with others in areas with high deprivation and are more likely to be single.

Among these UK adults with persistent seizures recruited from specialist clinics, their experience of having epilepsy was long, with a median of 18 years since diagnosis, and their epilepsy was difficult to control, with the majority (69%) experiencing 10 or more seizures in the previous year. Nearly half (45.8%) reported an additional history of a medical or psychiatric condition. Anxiety symptoms were twice as common as symptoms of depression (54 vs 28%). An even greater proportion of participants (63.1%) reported feeling to some extent stigmatized.

QoL measured by QOLIE-31-P had a mean score of 66 and varied widely over a range of 74 points. Factors that were associated with diminished total QOLIE-31-P were: depression, low self-mastery, anxiety, greater felt stigma, a history of medical and psychiatric comorbidity, low medication adherence, and greater seizure frequency, in decreasing order of effect.

### Generalizability of sample

A key strength of this study is that it recruited from a large group of PWE attending publicly funded epilepsy clinics, and results are likely to be generalizable to people in countries where medical care is also publically funded [26]. In such contexts, income is not a main factor in receiving access to health care. Studies from the USA find low QoL in epilepsy is associated with lower income [32]. We did not find that levels of deprivation were associated with QoL. Recruiting volunteers via advertisements or through user groups also requires an active response from patients, and may result in a patient group taking a more active role in managing their condition which may not be representative of the whole population [26]. An opt-out process can help with recruiting patients with memory problems, which can be a challenge to recruitment in epilepsy [35]. We had a relatively high recruitment rate at 37%, which is higher than a previous trial recruiting PWE [7]. Our current trial population does not represent the 60% of PWE without two or more seizures in the prior year, nor does it necessarily represent PWE with and without persistent seizures who are managed by doctors without referral to an epilepsy specialist. A small UK cohort study has found PWE recruited in primary care, at least 60% of whom would have epilepsy without persistent seizures, had a higher mean QOLIE-31 of 70 [18].

### Implications for clinical practice and research

Evidence from our UK group is consistent with evidence internationally that PWE and particularly those with persistent, frequent seizures have important psycho-social disadvantage and impaired QoL [8, 17, 19–21, 23, 24, 36]. Although our group had experienced epilepsy for a median of 18 years, these disadvantages have seemingly not been identified or, if identified, not redressed in usual medical care. Nevertheless, their QOLIE-31 scores were comparable to studies reported from other countries for people with and without persistent seizures. The UK group’s mean QOLIE-31 was 62 (SD 15.6) compared to a global mean score of 59.8 (SD 8.0) [17]. The wide range in UK mean QOLIE-31 scores (24.5–97.6) overlap the means reported in the lowest and highest scoring countries, the Russian Federation mean 42.1 (SD 4.1), and Canada mean 82 (SD 32.8), respectively [17].

Luoni et al. suggest that when epilepsy is accompanied by persistent seizures there is ‘a diagnostic gap’ when it comes to depression [36]. Screening for depression has been recommended [19, 20] but is still not routine. In epilepsy with persistent seizures, anxiety symptoms are even more common, and this is another diagnostic gap [37]. Many specialists work in isolation, with insufficient multi-disciplinary team support to address mental health issues, even if they are identified. If mental health issues are not redressed, there is likely over time to be a vicious cycle of negative consequences [3–5]. This requires exploration with longitudinal research, and development and testing of interventions to redress the gap. Research has begun on interventions aimed to reduce psychological distress, and because of their association, such interventions are more likely to affect QoL [38]. This research has not necessarily focused on people with poorly controlled epilepsy, who are likely to be most affected by psychological distress, and require intervention [10, 11].

Compared to other stigmatized conditions, there has thus far been less focus on testing interventions to reduce social impairment, such as stigma, lack of social support and lack of self-mastery in epilepsy care [8, 31]. Stigma, lack of social support and low self-mastery are potentially amenable to change. Moreover, it has been proposed that improving self-esteem and self-mastery is prerequisite if education is to lead to behaviour change [10, 31, 39]. In other stigmatized conditions, like HIV and mental ill-health, group interventions have been developed and tested specifically to provide social support, and to prevent isolation, loss of confidence, and self-stigma early on after diagnosis [40, 41]. People with epilepsy in the UK and elsewhere would benefit from this approach.

A question remains as to whether stand-alone educational interventions are likely to improve QoL. Memory problems are more prevalent among PWE with persistent seizures [25, 35]. Questions about memory are included in the cognitive subscale of the QOLIE-31 [22, 27], and so they may affect the total score. Memory issues are likely to make learning about self-management more challenging for PWE, with persistent seizures. Some studies report an improvement in epilepsy knowledge following self-management courses with follow-up at 6 months, at most [14, 42]. Long-term assessments have not been done, thus it is not known what impact memory would have on knowledge. A measure of acquired knowledge was not included in our study due to the volume of outcomes mandated by program funders. One hypothesis was that other measures, such as self-mastery, medication adherence and seizure frequency, could improve with increased knowledge. In the UK and other European countries, epilepsy nurses are taking an increasing role reinforcing advice about self-management [43–45] which could reduce the impact of memory issues. Self-management education, which is reinforced over time by a co-intervention of advice from a nurse or other professional, seems more likely to promote QoL [16, 43, 46].

## Conclusion

In common with PWE internationally, UK PWE reports impaired QoL compared to the general population and to PWE managed in primary care [17, 18]. The impairment in QoL in this large group of PWE with persistent seizures was significantly associated with symptoms of depression, lack of sense of self-mastery, anxiety, felt stigma and high seizure frequency, in diminishing order. Given this, stand-alone educational interventions may not be sufficient to change QoL. The close association of QoL and psychological distress supports a hypothesis that developing and testing stepped-up psychological interventions are more likely to improve QoL. In addition, social interventions, which aim to provide peer support and reduce stigma, may also improve QoL, especially early on after diagnosis [40, 41]. If psycho-social interventions result in more self-confidence, information derived from courses, provided as co-interventions or subsequently, may be more likely to promote QoL and self-management practice.
